# Applied Research for Better Disease Prevention and Control

**DOI:** 10.1371/journal.pntd.0003378

**Published:** 2015-01-08

**Authors:** Johannes Sommerfeld, Andrew Ramsay, Franco Pagnoni, Robert F. Terry, Jamie A. Guth, John C. Reeder

**Affiliations:** 1 Special Programme on Research and Training in Tropical Diseases (TDR), a co-sponsored programme of UNICEF/UNDP/World Bank/WHO, based at the World Health Organization, Geneva, Switzerland; 2 Global Malaria Programme, World Health Organization, Geneva, Switzerland; Dodowa Health Research Centre, Ghana

## Introduction

In 1976, two years before the Alma Ata Declaration, the then Director-General of WHO, Dr. Halfdan Mahler, proclaimed a “social revolution in public health” [1]. He saw the establishment of the Special Programme for Research and Training in Tropical Diseases (TDR) Programme in 1975 as playing an active part in the emerging social and health development agenda. In TDR's first public document, Mahler, and his UNDP counterpart Morse stated: “Many millions of the people living in the tropical regions of the world are cut off from the mainstream of social and economic progress. Victims of a heavy burden of disease as well as of harsh economic circumstances, they are not free to choose and plan a better future… Health and development are therefore inextricably interlinked and any strategy for improvement must be based upon this reality” [2].

In these early years of “international” health, it became increasingly apparent that the development of new health technologies was only one component of improved healthcare. Research was required to support the delivery of those technologies, as well as to understand the barriers that could prevent or limit access to them. TDR was one of the first United Nations co-sponsored programmes to recognize this need. From 1977 onward, it had a dedicated focus on applied social and economic research and produced a body of evidence that promoted and expanded the theoretical underpinnings of this field, supported training in low- and middle-income countries where the needs existed, and developed a research agenda and funding to specifically address the “social and economic factors that affect the transmission and control of disease… and to increase the effectiveness of disease control programmes by integrating human behavioural (social, cultural, and economic) factors in programme conception, design, and management” [3], [4].

In its first phase, research investigated human behaviour related to disease exposure, disease-related beliefs and attitudes, attitudes to disease control, the social and economic impact of tropical diseases, costs and effectiveness of alternative disease control measures, and economic development and disease [5]. It focused on the development of theory and methods for applying the social sciences in public health, community participation in disease control, and the economics of tropical diseases [6].

A later emphasis on the “upstream” factors in infectious diseases and their control, from 2000 to 2007, drew from basic social sciences and their subdisciplines, e.g., (medical) anthropology, (health) economics, (medical) sociology, political sciences, and health policy research [7]. Research focused on equity and access, health sector reform, community participation, gender-sensitive interventions [8], [9], globalization, human rights [10], and ethics [11], [12]. TDR also defined and exemplified the concept of applied social sciences for public health (ASSPH) [13], [14] and called for increasing the numbers of scientists trained in these areas. It identified implementation research as a critical aid to help disease control programmes, citing an “absence of capacity and understanding in how to engage with communities and ensure their participation, and of the ability to adapt research methods and health technologies to local contexts,” concluding that “the uptake, effectiveness, and sustainability of these interventions remains limited” [15].

Later, TDR led efforts to define and establish operational and implementation research as scholarly fields [16], [17]. Operational research was defined as “research into strategies, interventions, tools, or knowledge that can enhance the quality, coverage, effectiveness, or performance” of health systems or disease programmes [18]. Implementation research pursues research into the delivery of efficient, sustainable, and effective services; appropriate structure of health systems; the policy process; and other components that are necessary to bring new and old control interventions into the routine practice of national health systems and improve access particularly for vulnerable population groups. Both types of research are complementary [19].

## Implementing Programmes

From the late 1980s, TDR began to implement this growing body of research towards practical problems facing large disease control programmes. Many research and development (R&D) products were being used at large scale, yet the access was limited, particularly for the poor. The introduction of even simple interventions such as insecticide-treated nets (ITNs) revealed that an understanding of the social, cultural, and economic context is needed if such interventions are to be used effectively and the maximum public health benefit is to be achieved. Trials of ITNs in the mid-1990s demonstrated that scale-up was worthwhile and had a significant impact on reducing infant deaths from malaria [20]–[23].

Another important research effort showed how new treatments such as Artemisinin-based Combination Therapies (ACTs) and their unit-dose blister packs designed to be understood by illiterate populations improved the efficacy and delivery of antimalarial drugs, particularly by caregivers and trained members of the community [24]. The evidence for the effectiveness of community health workers (CHWs) to diagnose and manage malaria close to home, thus extending the healthcare system to the most remote and disadvantaged, was demonstrated in numerous studies that included the safe and effective distribution of ACTs [25], [26]. This work evolved into a broader Integrated Community Case Management strategy (iCCM) that provides care for malaria, pneumonia, diarrhoea, and other childhood illnesses and shows high levels of feasibility and impact on mortality [27]–[29].

In onchocerciasis control, following efficacy trials and its registration as a safe drug, ivermectin was ready for full-scale mass administration in the mid-1980s. Social research highlighted the issues of families being broken apart from the stigmatization due to the severe itching and disfiguration from the disease, which was just as important and disruptive to communities as the resulting blindness [30], [31]. Such findings on the psychosocial and economic impacts of onchocerciasis contributed to policy recommendations to establish the African Programme for Onchocerciasis Control (APOC) in 1995.

TDR's partnership with APOC led to several multicountry research initiatives that substantiated the effectiveness and sustainability of communities developing and managing annual treatment with ivermectin to control onchocerciasis [32], [33]. This was a significant change in how to manage mass drug treatment at the time, empowering communities and helping to establish new links to regional health centres.

APOC adopted this community-directed approach as its main strategy in 1996, and with TDR, has explored and proven its use for other diseases [34], such as malaria diagnosis and treatment, bednet distribution, and vitamin A supplementation to infants [35], [36].

Involving the community became more widely adopted through partnerships with the Global TB Programme, WHO regional and country offices, and an international partnership of investigators to ensure that implementation and operational research is used to improve tuberculosis control in low- and middle-income countries. A series of TDR-supported studies resulted in major changes to global policy recommendations on tuberculosis diagnosis between 2006 and 2012 [37], [38]. TDR has further advocated for the key role of implementation research in the introduction of new tools, the improvement of public health programmes, and the equitable delivery of services [16], [18], [19], [29], [39]. TDR has also helped facilitate the translation of evidence into policy and practice [37] by testing processes. This includes frameworks for assessing evidence for scale-up of new interventions.

Another important effort dealt with the elimination of visceral leishmaniasis from the sub-Indian continent [40]–[42] (cf. [43]). There are multiple strategies, but just one example highlights the value of implementation research, where trained community volunteers actively sought out those who could be infected so that treatment could be initiated earlier with a better outcome.

## Gender Research and Gender-Sensitive Interventions

Gender shapes the transmission dynamics, access to care, treatment, and effects in the body, leading to differential health outcomes among women and men. Gender-sensitive interventions are critical for the prevention and treatment of many tropical diseases. Since 1989, TDR has been addressing such issues through rigorous gender research [44], which began with a focus on women's issues and expanded to issues relating to both women and men [31]. The Task Force on Gender and Tropical Disease between 1994 and 1997 served as TDR's leading expert committee on gender-related issues and led to an extensive publication track record [45] and the definition of gender-responsive approaches in tuberculosis control [9] and in the rehabilitation of lymphatic filariasis [46].

## Addressing the Social and Environmental Determinants

Many of the infectious diseases grouped as “neglected” are caused and maintained by social and environmental determinants of health [47], and the challenge is to design and implement integrated, community-based prevention and control programmes addressing these determinants. TDR's *Global Report for Research on Infectious Diseases of Poverty* highlighted this area and called for more research on the interplay between demographic, social, and environmental factors in infectious disease occurrence [48]. Case studies on how public health programmes have addressed social and environmental determinants of health were published by TDR, as part of a WHO-wide knowledge network on priority public health conditions (PPHC-KN) in 2010 [49].

Since 2002, TDR has partnered with the Ecosystem and Human Health Programme of Canada's International Development Research Centre (IDRC) on multi-country research initiatives in Asia and Latin America to examine factors affecting dengue vector ecology and transmission and to tailor and test locally adapted and ecosystem-specific vector control interventions. This work is based on multidisciplinary research to identify links between health, the environment, and social determinants that might be harnessed more effectively for disease control, particularly vector control [50]–[52]. The research typically involves a situation analysis to characterize and map the urban ecosystem; vector ecology in its relation to rainfall; the social context, including stakeholder environment; and community dynamics, including gender implications. This process leads to the design of site-specific intervention packages using innovative biological, chemical, mechanical, and/or environmental vector control technologies and/or a combination of these tools [53], [54]. The model includes community participation, both from volunteers and government staff, and has led to changes in prevention practices in several countries. In 2012, TDR created a dedicated unit to continue this work. A new area within that unit now investigates the impact of vector-borne diseases in Africa and on population health vulnerabilities due to climate, environmental, and social change.

## Conclusions

Since its inception, TDR has helped to advocate for and built a solid track record in social, intervention, and implementation research applied to the prevention and control of infectious diseases of poverty. These diseases result from the complex interaction of biological, social, and environmental factors. They disproportionately affect poor and disadvantaged populations in which the poverty context reinforces risk and vulnerability. Control tools such as drugs, vaccines, and diagnostics often do not reach the populations that most need them because of social issues, because health systems fall short of potential, or because they are ill adapted to the cultural, social, and economic realities in which people live.

Research has evolved from addressing the social and economic determinants of health towards an approach where the community itself is key in developing a solution to those challenges. The work that TDR has supported has played a significant part in moving that understanding forward and has contributed to important results.

The model is evolving to meet current needs, such as with multidrug-resistant tuberculosis; with multidisease approaches such as for pneumonia, diarrhoea, and malaria; and for new environmental challenges, such as with climate change and its impact on vector-borne diseases. Today, there is a deeper array of research methods and tools that can be used to identify and implement solutions for the many diseases of poverty, solutions which TDR is committed to helping strengthen and expand. TDR's strategic framework for the years 2012–2017 places renewed emphasis on intervention and implementation research and on interdisciplinary research on vectors, environment, and society [55].
